# Editorial: Modeling neuromuscular diseases to determine molecular drivers of pathology and for drug discovery

**DOI:** 10.3389/fcell.2022.1017356

**Published:** 2022-10-05

**Authors:** Alec S. T. Smith, Megan L. McCain, Mark Bothwell, David L. Mack

**Affiliations:** ^1^ Department of Physiology and Biophysics, University of Washington, Seattle, WA, United States; ^2^ Institute for Stem Cell and Regenerative Medicine, University of Washington, Seattle, WA, United States; ^3^ Department of Biomedical Engineering, USC Viterbi School of Engineering, University of Southern California, Los Angeles, CA, United States; ^4^ Department of Stem Cell Biology and Regenerative Medicine, Keck School of Medicine, University of Southern California, Los Angeles, CA, United States; ^5^ Department of Bioengineering, University of Washington, Seattle, WA, United States; ^6^ Department of Rehabilitation Medicine, University of Washington, Seattle, WA, United States

**Keywords:** neuromuscular disease (NMD), disease modeling, iPSC (induced pluripotent stem cell), cell line (C2C12), therapy screening, *in vitro* versus *in vivo*

Recent advances in gene editing, organoid technologies, tissue engineering, and neuromuscular differentiation techniques from induced pluripotent stem cells (iPSCs) and other cell sources have produced great strides in modeling neuromuscular diseases. But which models are most appropriate for a given condition? Dogmatic allegiance to specific models that inadequately account for the complexity and/or heterogeneity of a given disease can hamper efforts to predict useful drug targets and screen novel compound efficacy. Perhaps what is needed is a greater willingness to critically appraise new models to understand what they do well, where they fall short, and how they can be leveraged, hand-in-hand, to gain a more holistic understanding of neuromuscular disease processes across spatial scales. This is the goal of our recent Research Topic presented in *Frontiers in Cell and Developmental Biology.* Here, we have collected manuscripts that detail recent advances in modeling neuromuscular tissues and diseases while simultaneously addressing the strengths, weaknesses, and design considerations that should be evaluated when deciding on the suitability of a given model for informing on disease etiology.

Feliciano et al. describe the use of CRISPR gene editing to create an iPSC-based model of Charcot-Marie-Tooth disease (type 2E). Neurons derived from this source recapitulate known pathologic phenotypes observed in patients, including the aberrant accumulation of neurofilament light chain protein in neuronal cell bodies. Inactivation of the diseased *NEFL* allele *via* gene editing ameliorates the disease phenotype observed in the dish, offering validation of allele-specific editing as a method to treat this peripheral neuropathy. This manuscript offers convincing evidence supporting the use of CRISPR and iPSC-derived neurons as models and screening platforms for inheritable peripheral neuropathic diseases. However, we invite readers to simultaneously consider data presented in Smith et al., which details how CRISPR editing and clonal expansion of iPSCs can lead to the emergence of potentially misleading phenotypes in stem cell-based models of ALS. This manuscript highlights the need to carefully consider data from multiple clones and lines generated using different editing techniques to gain confidence that the phenotype observed is due to the specific mutation in question and not an artifact of the history of a given cell line. By showcasing these papers together, we draw attention both to the potential for novel iPSC-based preclinical models to advance therapeutics as well as the possible difficulties associated with their use in identifying molecular drivers of pathology.

Our Research Topic also presents new culture technologies and discusses their potential for improving cell-based models of neuromuscular disease. Barthelemy et al. detail the use of micromolded hydrogel surfaces to enhance the differentiation and organization of myotubes reprogrammed from skin fibroblasts of muscular dystrophy patients. Such engineered substrates also extend the culture lifetime of patient-derived myotubes, enabling more comprehensive evaluation of patient-specific defects in myogenesis and responses to treatment by antisense oligonucleotides. A complementary approach used to pattern culture surfaces for neurons is presented by Capel et al. who demonstrate that aerosol jet printing can be used to develop intricate neuronal patterns on 2D surfaces. Integration of such complimentary technologies in the future may enable greater control of neuron-muscle interactions *in vitro* to better facilitate the study of neuromuscular function in health and disease. Alternatively, spatial organization of both muscle and neurons can be achieved using compartmentalized chambers, as demonstrated by Smith et al. Here, the authors developed functional neuromuscular junctions (NMJs) in a compartmentalized cell culture device and then examined the role of complement activation in contributing to NMJ functional decline in a cell-based model of Myasthenia Gravis.

The work of Barthelemy, Feliciano, and Smith, along with their co-authors, highlight that obtaining a cell type expressing a specific mutation may be insufficient to accurately model specific disease phenotypes. How the cells are maintained in culture and allowed to interact is of critical importance to ensure the collection of clinically-meaningful data. In support of this idea, we provide a review of design considerations for the development of multi-organ culture models by Malik et al. While not specifically focused on neuromuscular tissues, this review provides valuable discussion of design considerations for complex cell-based models, including organ scaling, vascular dimensions, and appropriate flow rates, all of which can significantly impact the translational potential of the output data.

Despite a great deal of focus on iPSCs in recent years, significant advances in neuromuscular biology continue to be made using immortalized cell lines. In particular, the C2C12 mouse myoblast line has been a driving force of *in vitro* muscle biology for decades. We include work by Shen et al. detailing the use of C2C12s to evaluate *Postn* inhibition as a treatment for myotonic dystrophy (type 1). The presented data demonstrate that *Postn* knockdown enhances myogenesis, potentially *via* modulation of the TGF-β/Smad3 pathway. Similarly, Barrett et al. use C2C12s to investigate the molecular mechanisms underpinning the development of proprioceptive intrafusal fibers in skeletal muscle. These studies illustrate how deeper elucidation of basic muscle biology contributes to identifying molecular drivers of pathologies and underscore that important insights can still be achieved using long-established neuromuscular cell lines that are generally more scalable and uniform than current iPSC derivatives.

The primary research presented in this Research Topic focuses on cell-based models of neuromuscular tissues, which is not intended to detract from the value and importance of *in vivo* models but largely reflects the expertise of the editors. To bring a more balanced perspective, we have invited reviews discussing the relative values and weaknesses of different *in vitro* and *in vivo* models of neuromuscular tissues and diseases. First, Fralish et al. provide a comprehensive overview of available NMJ models in the context of modeling a range of incurable neuromuscular disease states. Second, Hines et al. deliver a perspective on the development of integrative workflows combining human cell-based models with animals to elucidate the complex biology of aminoacyl tRNA synthetase mutant forms of Charcot-Marie-Tooth disease. Third, Hekmatnejad and Rudnicki review transplantation assays as tools for examining muscle stem cell (satellite cell) function *in vivo* as a means to improve transplantation efficiency in cell-based therapies for muscle diseases. Approaches that leverage the respective advantages of *in vitro* and *in vivo* models are likely to become a standard paradigm for neuromuscular disease modeling because they enable rigorous investigations from the molecular level to the complete organism.

The works collected here attempt to highlight recent innovations in neuromuscular disease modeling and re-emphasize that no single model offers the perfect platform to study the entirety of neuromuscular biology and disease. In fact, we must be critical and honest about the strengths and weaknesses of each model for the field to progress. By combining multiple preclinical models, we can obtain comprehensive and multi-scale phenotypic data that better predict which therapies have the greatest chances of clinical success. Hopefully, the ongoing development and adoption of innovative and integrative preclinical workflows will help increase the number of compounds succeeding during clinical trials and ultimately improve patient outcomes.

